# Cryptic species and parallel genetic structuring in Lethrinid fish: Implications for conservation and management in the southwest Indian Ocean

**DOI:** 10.1002/ece3.3775

**Published:** 2018-01-24

**Authors:** Amy J. E. Healey, Niall J. McKeown, Amy L. Taylor, Jim Provan, Warwick Sauer, Gavin Gouws, Paul W. Shaw

**Affiliations:** ^1^ Institute of Biological Environmental and Rural Sciences Aberystwyth University Aberystwyth Dyfed UK; ^2^ School of Biological Sciences Royal Holloway University Egham Hill Egham UK; ^3^ Department of Ichthyology and Fisheries Science Rhodes University Grahamstown South Africa; ^4^ South African Institute for Aquatic Biodiversity Grahamstown South Africa

**Keywords:** biodiversity hot spot, chaotic genetic patchiness, conservation, cryptic species, kinship, larval cohesion

## Abstract

Analysis of genetic variation can provide insights into ecological and evolutionary diversification which, for commercially harvested species, can also be relevant to the implementation of spatial management strategies and sustainability. In comparison with other marine biodiversity hot spots, there has been less genetic research on the fauna of the southwest Indian Ocean (SWIO). This is epitomized by the lack of information for lethrinid fish, which support socioeconomically important fisheries in the region. This study combines comparative phylogeographic and population genetic analyses with ecological niche modeling to investigate historical and contemporary population dynamics of two species of emperor fish (*Lethrinus mahsena* and *Lethrinus harak*) across the SWIO. Both species shared similarly shallow phylogeographic patterns and modeled historical (LGM) habitat occupancies. For both species, allele frequency and kinship analyses of microsatellite variation revealed highly significant structure with no clear geographical pattern and nonrandom genetic relatedness among individuals within samples. The genetic patterns for both species indicate recurrent processes within the region that prevent genetic mixing, at least on timescales of interest to fishery managers, and the potential roles of recruitment variability and population isolation are discussed in light of biological and environmental information. This consistency in both historical and recurrent population processes indicates that the use of model species may be valuable in management initiatives with finite resources to predict population structure, at least in cases wherein biogeographic and ecological differences between taxa are minimized. Paradoxically, mtDNA sequencing and microsatellite analysis of samples from the Seychelles revealed a potential cryptic species occurring in sympatry with, and seemingly morphologically identical to, *L. mahsena*. BLAST results point to the likely misidentification of species and incongruence between voucher specimens, DNA barcodes, and taxonomy within the group, which highlights the utility and necessity of genetic approaches to characterize baseline biodiversity in the region before such model‐based methods are employed.

## 1 INTRODUCTION

Understanding the evolution of marine biodiversity requires knowledge of the patterns and processes underpinning genetic structuring of species. Combined demographic–genetic studies can reveal the spatial and temporal scale at which evolutionary forces are occurring (Waples, 1998) and, by extension, identify cryptic components of biodiversity and so allow optimization of spatial conservation strategies. Identification of spatial and temporal processes is particularly important for harvested taxa, as genetic diversity and adaptation are key factors underpinning the resilience/sustainability, and ultimately the evolutionary potential, of populations and species (Iles & Sinclair, 1982; Ruzzante et al., 2006; Ryman, Utter, & Laikre, 1995; Therkildsen et al., 2013).

The southwest Indian Ocean (SWIO), which forms a subdivision of the tropical Indo‐Pacific, is renowned for its diverse marine habitats and resources, including an ichthyofaunal richness (Smith, Smith, & Heemstra, 2003) that supports significant artisanal, subsistence, and commercial fisheries across the region (Berg, Francis, & Souter, 2002). The complex oceanographic features of this region, characterized by the bifurcation of the South Equatorial Current into two continental currents and the series of eddies in the Mozambique Channel (Benny, 2002), are hypothesized to have contributed to this high biodiversity (Ridgway & Sampayo, 2005). Diverse patterns of genetic differentiation have been observed within species distributed across the SWIO, from high levels of gene flow (Chiang, Hsu, Wu, Chang, & Yang, 2008; Duda & Palumbi, 1999; Fratini, Ragionieri, & Cannicci, 2010; Muths, Grewe, Jean, & Bourjea, 2009; Muths, Le Couls, Evano, Grewe, & Bourjea, 2013; Ragionieri, Cannicci, Schubart, & Fratini, 2010; Silva, Mesquita, & Paula, 2010b) to more complex species‐specific patterns of isolation, including disruption of gene flow across the Mozambique Channel (Gopal, Tolley, Groeneveld, & Matthee, 2006; Muths et al., 2011; Silva, Mesquita, & Paula, 2010a; Visram et al., 2010) and isolation of the northern (Ragioneiri et al. 2010; Visram et al., 2010) and/or southern (Fratini & Vannini, 2002; Lessios, Kessing, & Pearse, 2001; Muths, Tessier, & Bourjea, 2015; Muths et al., 2011) Mascarene Islands from continental populations. Some investigations have found signatures of historical vicariance associated with sea‐level fluctuations during the Pleistocene (Ragionieri, Fratini, Vannini, & Schubart, 2009; Silva et al., 2010a). However, despite a growing body of ecological and evolutionary research the SWIO is still regarded as one of the least‐studied tropical ecosystems (Beheregaray, 2008; Ridgway & Sampayo, 2005).

The complex array of genetic population structures resolved to date within the SWIO highlight the potential hazards of making region‐wide predictions of population structure from single species. However, studies of ecologically similar and contemporaneously co‐distributed species represent analytical frameworks wherein variance between lineages, place, and time are minimized, and thus “general rules” informative to subsets of taxa may be resolved (Bird, Holland, Bowen, & Toonen, 2007, 2011; Bohonak, 1999; Dawson, 2012; Lester, Ruttenberg, Gaines, & Kinlan, 2007). In this study, we develop such an analytical framework by performing replicated genetic analysis of two lethrinid fish species: *Lethrinus harak*, the thumbprint emperor, and *Lethrinus mahsena*, the sky emperor. Both species are ecologically and biogeographically similar. The habitat of *L. harak*, the most abundant and commercially important lethrinid in the SWIO (Kulmiye, Ntiba, & Kisia, 2002), consists of shallow and protected coastal areas including reefs, mangroves, shallow lagoons, and seagrass beds (Ebisawa & Ozawa, 2009; Kulmiye et al., 2002). *Lethrinus mahsena* occupies similar shallow habitats but has been observed predominantly in reefs or adjacent areas (Carpenter & Allen, 1989). Like the majority of lethrinids, both species are relatively long‐lived (up to 15 years; Ebisawa & Ozawa, 2009) protogynous hermaphrodites (Ebisawa, 2006; Grandcourt, 2002), but many aspects of their reproductive biology are not known (Ebisawa, 2006; Kulmiye et al., 2002). Both species have pelagic larval stages with Nakamura et al. (2010) reporting a larval duration of around 29 days for *L. harak*.

The aim was to test the prediction that both species would display similar patterns of historical (phylogeographic) and contemporary population genetic structure across the SWIO. As the Lethrinidae is one of the most important families of fish for artisanal, recreational, and subsistence fisheries (Carpenter & Allen, 1989; Gouws, 2012), the genetic patterns are interpreted in the context of recurrent recruitment processes relevant to stock sustainability. To this end, kinship analyses were employed to complement estimates of connectivity/isolation based on traditional population genetic (e.g., *F*_ST_) methods. To provide an additional historical context, we combined genetic studies with ecological niche modeling (ENM), which has been shown to provide novel insights into the influence of historical biogeography on genetic variation in marine species (Glynn, Houghton, & Provan, 2015; Glynn et al., 2016).

## 2 MATERIAL AND METHODS

### 2.1 Sample collection and molecular analyses

**Figure 1:**
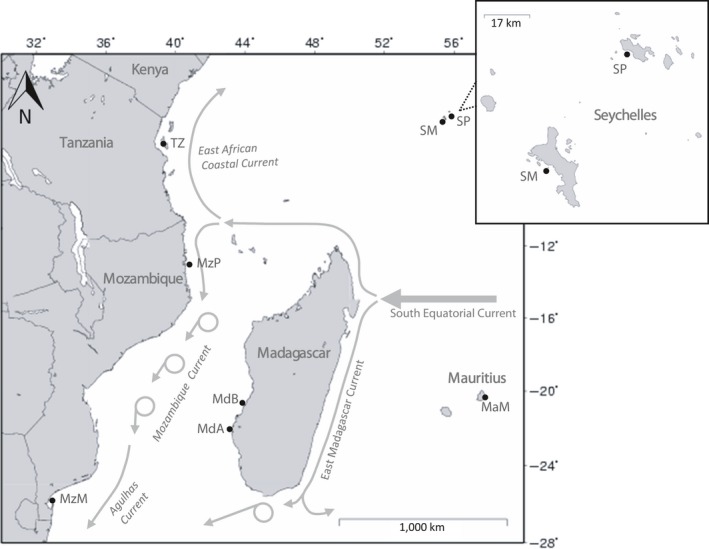
Sampling locations for *Lethrinus mahsena* and *Lethrinus harak* within the southwest Indian Ocean: Mauritius, Mahebourg (MaM); Seychelles, Praslin (SP); Seychelles, Mahe (SM); Tanzania, Zanzibar (TZ); Mozambique, Pemba (MzP); Madagascar, Belo sur Mer (MdB); Mozambique, Maputo (MzM)

Samples (fin clips fixed in 95% ethanol) were collected from commercial or subsistence catches landed in seven locations across the SWIO between 2009 and 2012 (Figure 1). Total DNA was extracted from all samples using a standard CTAB‐chloroform/isoamyl alcohol method (Winnepenninckx, Backeljau, & Dewachter,^1993^).

For both species, a fragment of the mtDNA cytochrome *c* oxidase subunit I (COI) gene was amplified by polymerase chain reaction (PCR) with species‐specific primers (*L. mahsena*: LmCOIF 5′‐TGGTAGGAACAGCCCTAAGC‐3′ & LmCOIR 5′‐ AGAATTGGGTCCCCTCCTC‐3′; *L. harak*: LhCOIF 5′‐CGAACTTAGTCAGCCCGGA‐3′ & LhCOIR 5′‐ TGCTGATAGAGGATTGGGTC‐3′) for a subset of individuals. PCRs were performed in a total volume of 20 μl, containing 4 μl template DNA, 2 mmol/L MgCl_2_, 0.5 μmol/L forward primer and 0.5 μmol/L of reverse primer, 0.2 mmol/L dNTP mix (20 μmol/L each dATP, dCTP, dGTP, dTTP), 1× reaction buffer [75 mmol/L Tris‐HCl, 20 mmol/L (NH_4_)_2_SO_4_], and *Taq* polymerase (BioTaq, 5 U/μl). The PCR thermoprofile was: 180 s at 95°C, followed by 45 cycles of 30 s at 95°C, 45‐s annealing at 55°C for both primer sets, and 60 s at 72°C, followed by a final 5‐min extension at 72°C. PCR products were then purified using EXOSAPIT and sequenced from both directions on an Applied Biosystems 3500 platform using the respective PCR primers. Sequences were aligned using the Clustal W (Thompson, Higgins, & Gibson, 1994) program, available in BioEdit (Hall, 1999), and analyzed using BLAST. Sequences revealed the presence of a highly divergent clade among the Seychelles *L. mahsena* samples (described in Section 2.1). To investigate the frequency of occurrence of this clade elsewhere, a diagnostic PCR‐RFLP method was developed and used to genotype all *L. mahsena* samples (Appendix S1: Figure S1).

Nuclear genetic variation was assessed at nine dinucleotide (100RTE, 95ACRTE, 95TGRTE, 90RTE, 80RTE, 75RTE, 68RTE, 58RTE, BST2.33) and one trinucleotide (96RTE) microsatellite loci described by Van Herwerden, Benzie, Peplow, and Davies (2000); Van Herwerden, Benzie, and Davies (2003). PCRs were performed under the following conditions: 180 s at 95°C, followed by 35 cycles of 30‐second denaturing at 95°C, 30‐s annealing at 50°C, and 30 s at 72°C. In order to increase product strength and reduce nonspecific products, PCR protocols were altered for a subset of loci. Annealing temperature was increased to 60°C for 96RTE and 95ACRTE. For 75RTE, 68RTE, and 80RTE, the number of cycles was increased to 55 and annealing temperatures increased to 60°C, 55°C, and 54°C, respectively. Each 10 μl reaction contained 3 μl template DNA, 1 pmol of each primer, 5 μl of 2xBioMix [1.5 mmol/L MgCL2] (Bioline, UK), and 1 μl of ddH_2_O. Amplicons were separated on an Applied Biosystems 3500 with alleles inferred using the Peak Scanner software (Applied Biosystems). To ensure robustness of genotypes, we employed a double genotyping as described by McKeown, Arkhipkin, and Shaw (2017).

### 2.2 Statistical analysis of mtDNA sequences

After pruning sequences, a total of 486 bp of the mtDNA COI gene was aligned across 72 *L. mahsena* and 104 *L. harak* samples. All analyses of mtDNA sequences were conducted in Arlequin 3.5.1.2 (Excoffier & Lischer, 2010) unless otherwise stated. Phylogenetic relationships among sequences were inferred using maximum likelihood (ML) trees in MEGA v6.06 (Tamura, Stecher, Peterson, Filipski, & Kumar, 2013) and Bayesian inference performed using MrBayes v3.2 (Ronquist & Huelsenbeck, 2003). For both species, the K2P + G + I substitution model, identified as optimal using Modeltest 3.7 (Posada & Crandall, 1998), was used. Maximum likelihood bootstrap values were calculated using 1000 bootstrap replicates, and Bayesian inference (BI) was calculated assuming unknown model parameters, and run over 5,000,000 generations, sampling the Markov chain every 1000 generations, with the first 15% of trees discarded as burn‐in. Median‐joining haplotype networks were constructed in Network 5.0 (Fluxus Technology). Percentage sequence divergence within and between species/clades and K2P distances were calculated using MEGA v6.06.

Genetic variation was assessed using calculations of the number of haplotypes (*H*), in addition to indices of haplotype (*h*) and nucleotide (π) diversity (Nei, 1987) alongside their variances. Genetic differentiation among samples was further tested by global and pairwise Φ_ST_ using pairwise haplotype distances (Weir & Cockerham, 1984), with associated *p* values estimated after 10,000 permutations.

### 2.3 Statistical analysis of microsatellite data

The number of alleles (NA), allelic richness (*AR*), and observed (*H*_O_) and expected (*H*_E_) heterozygosities were calculated using GENALEX 6 (Peakall & Smouse, 2006). Deviations from Hardy–Weinberg (HW) expectations and linkage disequilibrium between pairs of loci were assessed using exact tests in Genepop v4.2 (Raymond & Rousset, 1995).

Genetic differentiation was quantified using global and pairwise *F*‐statistics (Wright, 1978). To account for possible null allele effects, such indices were also calculated using the null alleles adjustment in FreeNA (Chapuis & Estoup, 2007). To test for signatures of isolation by distance (IBD), correlations between geographical distances (minimum sea distance in nautical miles) and linearly transformed genetic differences (*F*_ST_/(1 − *F*_ST_) Slatkin, 1993) were tested using a MANTEL matrix correlation test in GENALEX. Genetic structure was also investigated using Bayesian “group assignment” “without admixture” methods implemented in BAPS 6.0 (Corander, Waldmann, Marttinen, & Sillanpaa,^2004^), for models of *K* = 1–6 (10 independent runs per K). Genetic relationships among samples were visualized using factorial correspondence analysis (FCA) in GENETIX (Belkhir, Borsa, Chikhi, Raufaste, & Bonhomme, 2004). Additionally, to assess nuclear differentiation between descendants of the two highly divergent mtDNA clades observed in *L. mahsena* (Seychelles), self‐classification tests (i.e., assignment of nuclear genotypes to clade defined baseline groups) were employed in GENECLASS 2 (Piry et al.,^2004^). The chord genetic distance (Cavalli‐Sforza & Edwards, 1967) was used due to the deviations from Hardy–Weinberg equilibrium.

Mean pairwise relatedness within samples was calculated using the relatedness estimator *r*_qg_ of Queller and Goodnight (1989) in GENALEX (Peakall & Smouse, 2006) with associated 95% confidence intervals determined by 1,000 bootstraps. Permutation of genotypes among all samples (999 times) was used to calculate the upper and lower 95% confidence intervals for the expected range of *r*_qg_ under a panmictic model. The maximum likelihood method implemented in ML‐RELATE (Kalinowski, Wagner, & Taper, 2006) was used to infer the relationships among pairs of individuals, specifically to categorize them as unrelated (U), half‐sib (HS), full‐sib (FS), or parent offspring (PO). Pairs of individuals were classified into three categories: (a) unrelated (classification as U only), (b) related (classification as any combination of HS, FS, PO but not U), or (c) ambiguous (classification as U as well as some related state).

### 2.4 Estimation of Type I and Type II error rates

To assess differences in statistical power among the data sets for both species and marker types, Type I and Type II error rates were estimated using POWSIM (Ryman & Palm, 2006). Analyses were run for a combination of the average and smallest sample sizes for each species (adjusted for mtDNA following Larsson, Charlier, Laikre, & Ryman, 2009).

### 2.5 Paleodistribution modeling

To evaluate suitable habitat ranges (and any geographical range changes) of *L. harak* and *L. mahsena* during the Last Glacial Maximum (LGM; ~21 KYA), paleodistribution modeling was conducted. Post‐1950s species occurrence data were obtained from the Global Biodiversity Information Facility (GBIF; http://www.gbif.org), along with the Ocean Biogeographic Information System (http://www.iobis.org), resulting in a total of 335 occurrences of *L. harak* and 152 of *L. mahsena*. Contemporary bioclimatic data, specifically generated for marine environments, were collected at 5‐min resolution from MARSPEC (Sbrocco & Barber, 2013; Table S1). Models were generated in MAXENT v3.3.3 (Phillips, Anderson, & Schapire,^2006^) and cross‐validation conducted using 10 replicate runs, under the default parameters. Model performance was assessed based upon the area under the receiver operating characteristic curve (AUC). Finally, the models were projected onto bioclimatic data reconstructed to represent the LGM (Sbrocco, 2014), using an ensemble of five different models (CNRN, ECBILTCLIO, FGOALS, Had‐CM, and MIROC‐322).

## 3 RESULTS

### 3.1 Cryptic genetic divergence in sympatry

**Figure 2:**
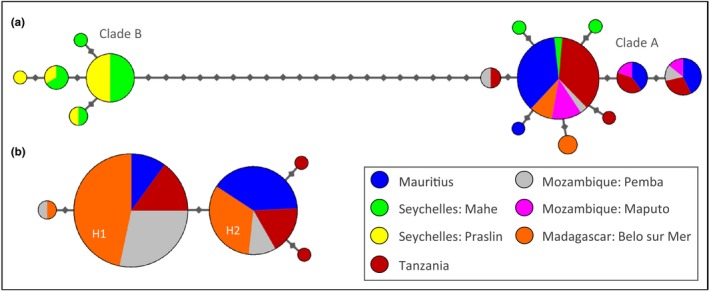
Haplotype network of (a) *Lethrinus mahsena* and (b) *Lethrinus harak* based on 486 bp of COI mtDNA. Each small gray diamond represents a single mutation; branch lengths are proportional to the number of differences, and node size is proportional to the haplotype frequency

Phylogenetic reconstruction of COI haplotypes among putative *L. mahsena* revealed the presence of two highly divergent clades within the SWIO, which were separated by 20 mutational steps (Figure 2). Diagnostic PCR‐RFLP analysis of all samples (Appendix S1) confirmed that clade A individuals occurred across all samples (Mauritius, Mahe Island Seychelles, Tanzania, Mozambique Pemba, Mozambique Maputo, Madagascar Belo sur Mer) excluding Praslin Island Seychelles, whereas clade B individuals were found in the Seychelles samples, where they co‐occurred with, and were more frequent than, clade A fish.

**Figure 3:**
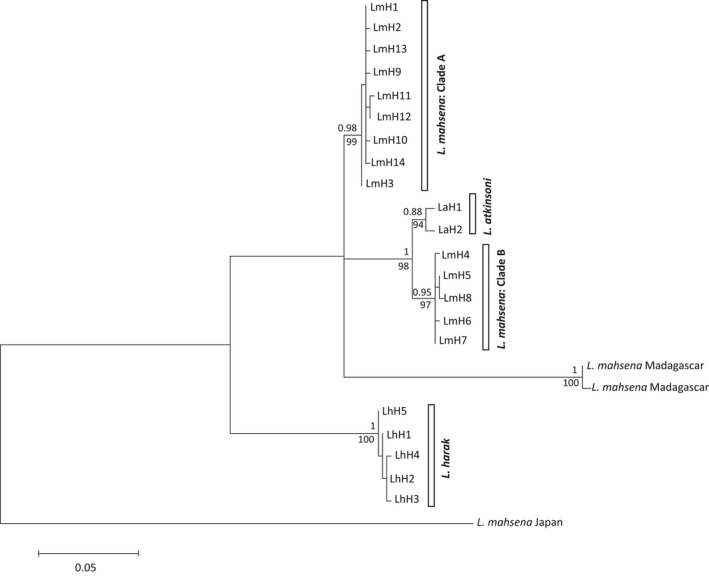
Phylogenetic relationships within *Lethrinus* species using 486 bp COI mtDNA. Statistical support for nodes is given for both Bayesian analyses (posterior probabilities) above branches and ML analyses (bootstrap support) below branches. Branch tips are labeled as such: Lm = *Lethrinus mahsena*, La = *Lethrinus atkinsoni*, Lh = *Lethrinus harak*. Haplotype (*H*) numbers for *L*. mahsena and *L. harak* correspond to Tables S2 and S3, for *L. atkinsoni* H1 =  GenBank sequences from Queensland, Australia (KP194639.1, KP194307.1). laH2 =  GenBank sequences from the Philippines (KF009603.1, KC970391.1). *L. mahsena* Madagascar and *L. mahsena* Japan = sequences deposited as *L. mahsena* on GenBank (JQ350088.1, JQ350089.1, JF952782.1) that do not correspond phylogenetically to this classification

**Figure 4:**
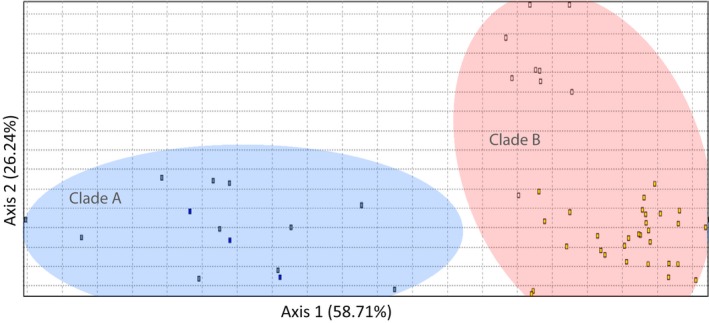
Factorial correspondence analysis showing multivariate relationships among microsatellite genotypes of *Lethrinus mahsena* samples from Mahe (SM) and Praslin (SP) Islands in the Seychelles. Samples were sorted by mitochondrial CO1 clade (clade A/clade B), classified by through PCR‐RFLP analysis

BLAST searches of clade A individuals revealed a 99% sequence similarity to *L. mahsena* voucher specimens from Mozambique (JF493750.1, JF493751.1, JF493752.1); however, BLAST searches of clade B individuals also identified 99% sequence similarity to *L. mahsena* voucher specimens from India (EF609387.1, KM079305.1, KM079304.1, KJ920117.1); this was followed by 97% sequence similarity to all available *L. atkinsoni* voucher specimens from Western Australia, the Philippines, and Taiwan (KV944053.1, KP194639.1, KP194152.1, KP194307.1, KF009603.1, KC970391.1, EF609384.1). The average sequence divergence between clade A and clade B was 4.6% (k2p distance = 0.048, *SE* = 0.010; Table S4) with clade B less divergent from *L. atkinsoni* (2.2%; k2p distance = 0.021, *SE* = 0.006; Figure 3). Microsatellite genotypes revealed a clear differentiation between individuals partitioned according to their mtDNA clade in the FCA (Figure 4), with a corresponding high *F*_ST_ (0.01, *p* < .001). This pattern was also supported by assignment tests which found complete self‐classification to clade with the exception of a single individual from clade A, which had a similar probability of assignment to both clades (0.55 and 0.45 assignment probability to clade A and B groups, respectively).

### 3.2 mtDNA COI sequence data

Overall haplotype and nucleotide diversities (Table 1) were similar for both *L. harak* (*h* = 0.52, π = 0.0011) and *L. mahsena* (*h* = 0.59, π = 0.0018). Network representation of *L. mahsena* (excluding clade B) revealed a single haplogroup dominated by a single common haplotype present across all SWIO localities. Similarly for *L. harak*, a single haplogroup was dominated by two common haplotypes (Figure 2): haplotype 2 which was dominant in Mauritius and haplotype 1 which was dominant across all other SWIO localities.

**Table 1:** (a) Mitochondrial genetic diversity levels, mtDNA neutrality tests, and a summary of mismatch distributions for 486 bp of *Lethrinus harak* and *Lethrinus mahsena* COI mtDNA: mtDNA sample size = *N*, number of haplotypes = *H*, number of private haplotypes = pHap, haplotype diversity = *h*, nucleotide diversity = π. (b) Microsatellite genetic diversity across nine (*L. harak*) and 10 (*L. mahsena*) loci in *L. harak* and *L. mahsena,* respectively: microsatellite sample size = *N*, number of alleles = *N*_A_, expected heterozygosity = *H*_E_, observed heterozygosity = *H*_O_, and probability of deviation from Hardy–Weinberg expectations = *p*. For both mitochondrial and microsatellite data sets statistically significant estimates (*p* < .05) are highlighted in bold and instances where tests could not be performed are indicated as such (−)

	(a) mtDNA (COI)					(b) Microsatellite				
	*N*	*H*	Phap	*h*	π	*N*	*N*_A_	*H*_O_	*H*_E_	*p*
Mauritius										
* L. harak*	22	2	0	0.42 (0.09)	0.0008 (0.0009)	43	4.00	0.52	0.45	**.000**
* L. mahsena*	18	4	1	0.54 (0.12)	0.0017 (0.0014)	19	4.40	0.59	0.56	**.023**
Seychelles Mahe										
* L. mahsena*	3	3	2	1.00 (0.27)	0.0027 (0.0028)	12	3.60	0.58	0.50	**.044**
Tanzania										
* L. harak*	18	4	2	0.63 (0.07)	0.0015 (0.0013)	60	4.11	0.42	0.42	**.000**
* L. mahsena*	18	5	1	0.57 (0.13)	0.0016 (0.0014)	21	4.40	0.54	0.53	.412
Mozambique Pemba										
* L. harak*	22	3	0	0.39 (0.11)	0.0008 (0.0008)	44	3.89	0.41	0.42	**.000**
* L. mahsena*	3	3	0	1.00 (0.27)	0.0041 (0.0039)	7	3.10	0.46	0.45	.246
Mozambique Maputo										
* L. mahsena*	6	3	0	0.60 (0.22)	0.0018 (0.0020)	10	3.60	0.45	0.49	.053
Madagascar Belo sur Mer										
* L. harak*	42	3	0	0.47 (0.06)	0.0009 (0.0009)	100	4.78	0.42	0.42	**.000**
* L. mahsena*	5	2	1	0.60 (0.18)	0.0012 (0.0014)	5	2.30	0.53	0.40	.991
Total										
* L. harak*	104	5	2	0.52 (0.03)	0.0011 (0.0019)	247	4.19	0.44	0.42	**.000**
* L. mahsena*	53	9	7	0.59 (0.07)	0.0018 (0.0036)	72	3.57	0.52	0.49	**.025**

Although global values of genetic differentiation were high and significant for *L. harak* (Φ_ST_ = 0.151, *p *=* *.002), pairwise tests revealed all significant values to be associated with two samples (Mauritius and Mozambique Pemba), with only the comparison between Mauritius and Mozambique Pemba remaining significant after Bonferroni correction (Table 2). Global differentiation was lower and not significant for *L. mahsena* (Φ_ST_ = 0.024, *p *=* *.277), and although pairwise Φ_ST_ was high in many cases, they were all nonsignificant (Table 2).

**Table 2:** Pairwise estimates of genetic differentiation (*F*_ST_) between *Lethrinus mahsena* samples (below diagonal—across 10 microsatellite loci corrected for null alleles (msat) and 486 bp of mtDNA COI) and *Lethrinus harak* samples (above diagonal—across nine microsatellite loci corrected for null alleles (msat) and 510 bp mtDNA COI)

	Mauritius	Seychelles Mahe	Tanzania	Mozambique Pemba	Mozambique Maputo	Madagascar Belo sur Mer
Mauritius						
COI			0.049	0.407*		**0.256**
msat			0.039*	0.042*		0.029*
Seychelles Mahe						
COI	0.152					
msat	0.121*					
Tanzania						
COI	−0.038	0.127		**0.127**		0.035
msat	0.043*	0.241*		0.022*		0.012*
Mozambique Pemba						
COI	−0.044	0.000	−0.043			0.002
msat	0.118*	0.128*	0.239*			0.013*
Mozambique Maputo						
COI	−0.116	0.100	−0.092	−0.154		
msat	0.194*	0.191*	0.289*	0.075*		
Madagascar Belo sur Mer						
COI	0.176	0.157	0.150	0.164	0.178	
msat	0.065*	0.241*	0.088*	0.218*	0.267*	

Statistically significant estimations (*p *<* *.05) are indicated in bold, and those significant after Bonferroni correction are denoted with a *.

Owing to small sample sizes, demographic tests were conducted globally. Neither Tajima's *D* (*D *=* *−0.524, *p *=* *.335) nor Fu's *Fs* (*Fs* = −1.100, *p *=* *.280) tests detected global deviations from neutrality for *L. harak*. For *L. mahsena* Fu's *Fs* was significant (*Fs *= −4.617, *p = *.002), but Tajima's *D* was not (*D *=* *−1.330, *p *=* *.070). Mismatch analyses were compatible with expansions for *L. mahsena* (τ = 1.078, 95% CI: 0–3.43, *p *=* *.690) but not in *L. harak* (τ = 0.723, 95% CI: 0.473–1.076, *p = *.010).

### 3.3 Microsatellite allele frequency data

A total of 247 individuals of *L. harak* from four sample locations and 72 *L. mahsena* individuals (excluding those belonging to clade B) from six sample locations were screened across nine and 10 microsatellite loci, respectively. Levels of intrasample genetic variability were similar in both species (Table 1). No significant linkage disequilibrium was indicated between any pair of loci in either species. POWSIM analysis indicated that microsatellite data conferred more power to detect genetic differences than mtDNA, with a similar resolution for both species (Table S5), and with generally low Type I error probabilities.

For *L. mahsena,* eight of 60 locus by sample tests revealed significant deviations from HWE (Table S6), primarily due to heterozygote deficits spread relatively consistently across five loci (80RTE, BST2.33, 100RTE, 95TGRTE, and 68RTE). For *L. harak*, 12 of 36 tests demonstrated significant deviations from HWE (Table S7), again largely due to heterozygote deficits with locus 58RTE exhibiting a deficit of heterozygotes in all four samples.

**Figure 5:**
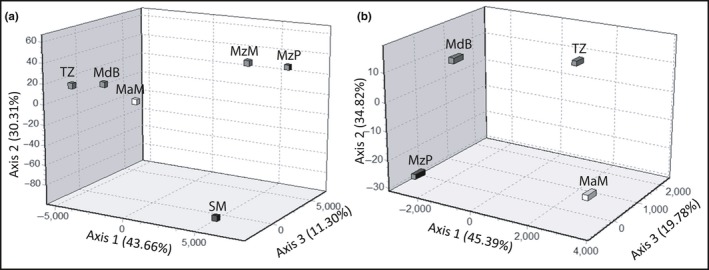
Factorial correspondence analysis showing multivariate relationships among microsatellite genotypes of SWIO samples of (a) *Lethrinus mahsena* and (b) *Lethrinus harak*

Global estimates of differentiation (with null allele correction) were higher for *L. mahsena* (*F*_ST_ = 0.164) than for *L. harak* (*F*_ST_ = 0.023) but highly significant in both cases. Pairwise *F*_ST_ estimates were also high and significant between all samples for both *L. harak* and *L. mahsena* (Table 2). No significant IBD effects were detected for either species [*L. mahsena, R* ^2^ = .071, *p* = .340; *L. harak, R* ^2^ = .434, *p* = .090]. BAPS analysis detected two genetically distinct groups within the *L. harak* data set, with Mauritius separated from the other SWIO samples (Figure S1); however, FCA divided samples into four groups corresponding to sampling sites (Figure 5). For *L. mahsena*, BAPS analysis identified three groups with samples from the Seychelles, Mozambique, and the rest of the SWIO each appearing to fall within distinct populations (Figure S1) supported by FCA (Figure 5).

Values of relatedness based on r_gq_ fell within the 95% confidence limits expected for panmictic populations for all samples of *L. harak* (Figure S2); however, mean values greater than expected under panmixia were identified in *L. mahsena* (Figure S3). Maximum likelihood inferences of relatedness among pairs of individuals (Figures S4 and S5) identified high levels of ambiguous kinship inference (i.e., individuals could not be unambiguously classified as related or unrelated for both *L. harak* (88.54%) and *L. mahsena* (79.56%)) but did identify related dyads within all samples for both species except Madagascar, Belo sur Mer for *L. mahsena*.

### 3.4 Paleodistribution modeling

**Figure 6:**
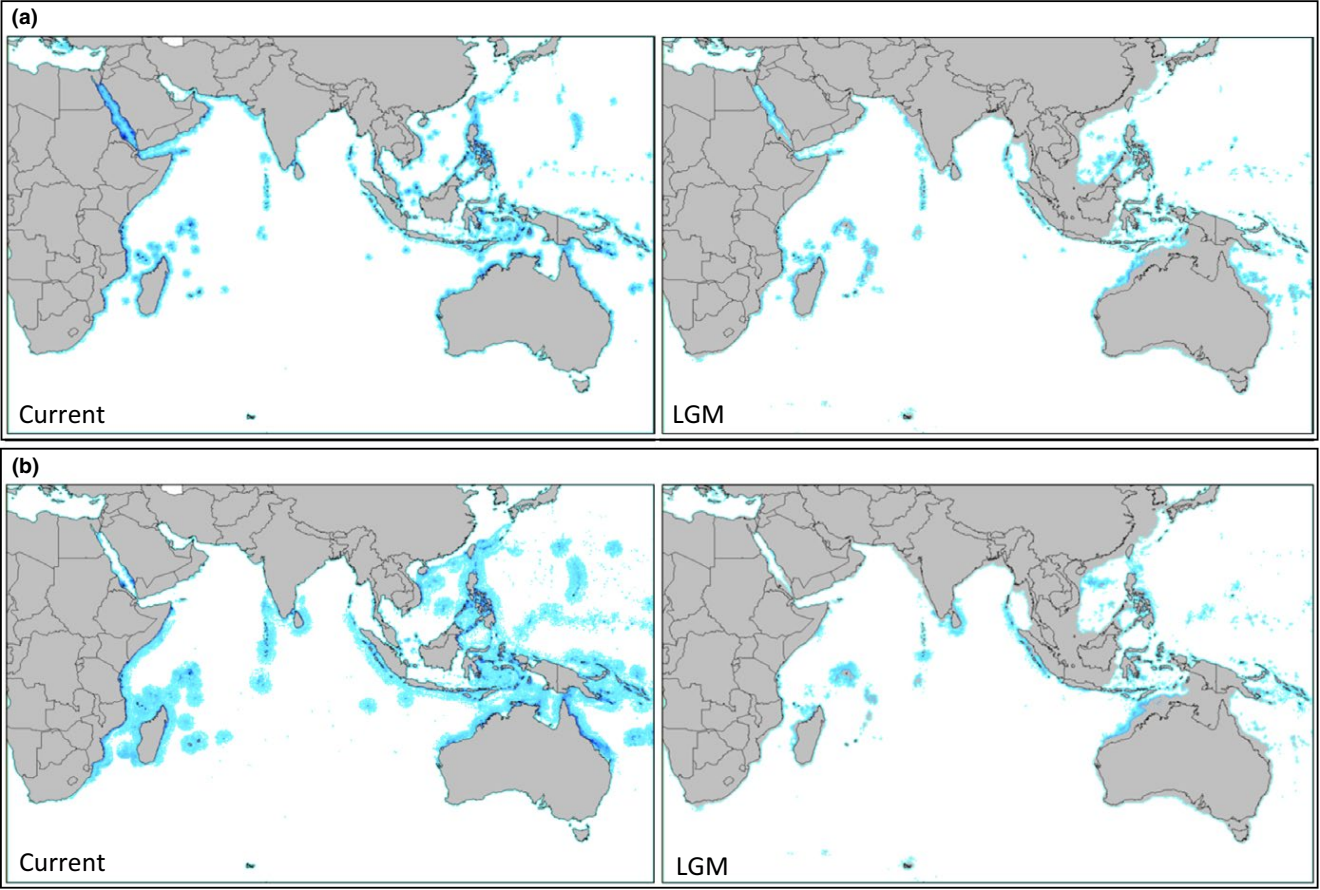
Results of species distribution models depicting predicted ranges of (a) *Lethrinus mahsena* and (b) *Lethrinus harak* during the present day and the Last Glacial Maximum (LGM, ~21KYA)

Mean AUC values were 0.985 (*SD* = 0.003) and 0.980 (*SD* = 0.016) for *L. harak* and *L. mahsena*, respectively, indicating very good model performance. Present‐day models for both *L. harak* and *L. mahsena* appear to accurately reflect the known contemporary ranges of both species, with suitable habitats aggregated in coastal environments across the Indian and western Pacific Oceans (Figure 6). Despite an overall reduction in suitable habitat during the LGM, this appears to take the form of a geographically homogeneous contraction across both species ranges rather than obvious vicariant fragmentation of present‐day ranges (Figure 6).

## 4 DISCUSSION

### 4.1 Cryptic sympatric species

An important result was the detection of two highly divergent mtDNA clades (A and B) co‐occurring within samples all described initially as *L. mahsena* from two sites in the Seychelles. Targeted PCR‐RFLP analysis of all of our samples suggests that clade B was confined to the Seychelles samples. Although both clade A and clade B exhibited high sequence similarity with *L. mahsena* voucher specimens from Mozambique and India, respectively, sequence divergence between the two clades (mean 4.4%) was in excess of even conservative thresholds of species‐level divergence (Roux et al., 2016; Zemlak, Ward, Connell, Holmes, & Hebert, 2009). Microsatellite analysis also revealed a significant nuclear differentiation among members of the two clades, with biparentally restricted gene flow also apparent in results of assignment tests (high levels of assignment to clade). Clade B sequences were more similar to sequences of *L. atkinsoni*, a species described from west Pacific waters but not in the WIO. Sequence divergence between clade B and *L. atkinsoni* (mean = 2.2%; K2P distance = 0.021) is in the gray zone of the species divergence continuum (Roux et al., 2016), suggesting that clade B may represent a highly divergent *L. atkinsoni* clade lying well outside of the currently described species distribution, or that clade B represents an undescribed species most closely related to *L. atkinsoni*. Overall, the cytonuclear differentiation indicates the sympatric occurrence of a second species alongside *L. mahsena*, which is being indiscriminately harvested as *L. mahsena*. Furthermore, the data point to incongruence between current taxonomy and DNA barcodes. Specimens from India belonging to this potential cryptic species may have been misidentified as *L. mahsena* and used as voucher specimens. Further evidence of misidentification is apparent among sequences deposited as *L. mahsena* on GenBank: those from Madagascar (JQ350088.1, JQ350089.1) have a greater similarity (99%) with *L. lentjan* voucher specimens from Iran, whereas supposed *L. mahsena* sequences from Japan (JF952782.1) exhibit greatest similarity (91%) *to L. rubrioperculatus*. In general, the meristic and morphological features of emperor fish are conservative (Carpenter & Allen, 1989) and distinct species are often difficult to identify easily by morphology alone (Sato, 1971; Smith, 1959). Failure to describe the full species diversity and/or misidentification of species may fundamentally compromise conservation strategies and tools. For example, the ENM performed here was based on reported occurrences of the species; however, based on the genetic results such occurrence data may be inaccurate. By extension, the modeled habitat patches may actually reflect distinct species ranges. The cryptic biocomplexity resolved here, as well as other cases of identification of cryptic lethrinid species (Borsa, Hsiao, Carpenter, & Chen, 2013), emphasizes the need for genetic studies on a wider geographical scale to fully resolve levels of intraspecific and interspecific diversification, as well as for the development of low‐cost species identification and assignment techniques as resources for management such as the PCR‐RFLP procedure developed here.

### 4.2 Population history of *Lethrinus harak* and *Lethrinus mahsena* across the SWIO

Hindcasting of ecological niche models (ENMs) suggested a marked reduction in suitable habitat for both *L. mahsena* and *L. harak* during the LGM that would be expected to have decreased their range‐wide population sizes. Therefore, as for many other SWIO taxa including numerous reef‐associated fish (Craig, Eble, Bowen, & Robertson,^2007^; Visram et al. 2010) and crustaceans (Fratini et al.,^2010^; Gopal et al.,^2006^; Tolley, Groeneveld, Gopal, & Matthee,^2005^), it is likely that eustatic sea‐level fluctuations during the Pleistocene effected demographic changes in SWIO lethrinids. mtDNA phylogenies for both species conformed to classical “star”‐shaped patterns (Slatkin & Hudson, 1991) expected under population expansion models. Demographic tests also provided some support for population expansion events for *L. mahsena* but not *L. harak*; however, the resolution of these tests may have been limited by a combination of low numbers of informative sites and differing sample sizes. The lack of genetic breaks within the mtDNA phylogenies for both species is compatible with a lack of prolonged vicariance. Similarly shallow phylogenies have been reported in many other SWIO species. Therefore, it would appear that for many species there has been little phylogeographic diversification within the SWIO (Hoareau, Boissin, & Berrebi,^2012^; Muths et al.,^2015^) with the majority of cases of deep phylogeographic structure within the region due to colonization of allochthonous lineages (Ragionieri et al., 2009, 2010; Silva et al., 2010a).

### 4.3 Population structure of *L. harak* and *L mahsena* across the SWIO

Although mtDNA Φ_ST_ values were high for both species, pairwise comparisons between samples were largely nonsignificant. In contrast, highly significant nuclear (microsatellite) differentiation was reported among samples for both species. Such a pattern is compatible with the greater statistical power of the nuclear data set as estimated by POWSIM. Similarly, Muths et al. (2011) detected a significant fine scale genetic structuring in the WIO for *Myripristis berndti,* while a previous mtDNA‐based study indicated extensive connectivity (Craig et al., 2007). For both *L. harak* and *L. mahsena,* almost all pairwise tests (*F*_ST_ and exact) were significant, including comparisons between nearby samples, with no clear geographical pattern or significant IBD (there was also no geographical pattern to the mtDNA φ_ST_ values). The structuring for both species was also significant upon conservative correction for null alleles (as discussed by Shaw et al., 2010) and was not driven by single‐locus effects (no loci identified as outliers). *F*_ST_ values were considerably higher for *L. mahsena* than for corresponding comparisons in *L. harak*. While POWSIM indicated a low Type I error rate for both data sets in qualitative (allele frequency) tests, the smaller sample sizes for *L. mahsena* may have inflated *F*_ST_ values (Willing, Dreyer, & Van Oosterhout, 2012). From a biological viewpoint, the considerably greater abundance of *L. harak* may also point to smaller effective population sizes for *L. mahsena* which could predispose the species to greater genetic drift effects and hence larger *F*_ST_ even if migration rates are similar (Whiteley, Spruell, & Allendorf, 2004). Further insight into the drivers of genetic differentiation was provided by the kinship analyses, which examine how alleles are shared among individuals and provide an independent test of the hypothesis that structure as quantified by *F*_ST_, which focuses on population allele frequencies, is a result of connectivity (Christie, Johnson, Stallings, & Hixon, 2010; Iacchei et al., 2013). Firstly, for *L. mahsena* mean kinship values for all samples exceeded those predicted within a nonstructured system. Second, related individual pairs and/or potentially related dyads (i.e., dyads that could not be unambiguously described as unrelated) were found in all samples for both species. While the large number of dyads that could not be unanimously classified to a single relationship category (i.e., unrelated or related) highlights the resolution threshold of the data, the results for both allele frequency and allele‐sharing analyses indicate that, even though sample sizes are small in some cases, the genetic heterogeneity cannot be dismissed as statistical noise but rather reflects some changes in the composition that may be a useful tool for better understanding recruitment dynamics and connectivity in these species (Knutsen et al., 2011; Selkoe, Gaines, Caselle, & Warner, 2006).

The geographically patchy genetic structuring and kinship patterns could be generated by three nonmutually exclusive processes: population isolation, large variances in individual reproductive success (sweepstakes recruitment), and limited mixing of larvae from genetically different sources (larval cohesion). Facets of the genetic structure exhibited some congruence with known biogeographic boundaries and patterns of population isolation inferred from other species. For *L. harak*, there was pronounced differentiation of the Mauritian population from the rest of the SWIO, supported by clustering and pairwise *F*_ST_ > 0.03 in all comparisons. Although not resolved in the clustering analysis, the Mauritius *L. mahsena* sample was also highly differentiated from all other samples (pairwise *F*_ST_ values > 0.04). Differentiation of Mascarene populations has been observed for several other reef‐associated species (Muths et al.,^2011^,^2015^) and linked to a combination of large geographical distances from other coasts (>1,000 km), and a barrier effect due to the landmass of Madagascar. Genetic differentiation of the *L. mahsena* Seychelles sample was also supported by pairwise tests and clustering analysis. A combination of the South Equatorial Current and Equatorial Counter Current could serve to restrict connectivity between more western/southern locations as suggested by Muths et al. (2015). Such an isolating mechanism might also explain the seeming absence of clade B from other SWIO sites. The high fecundity and batch spawning behavior of both species could be conducive to sweepstakes effects, while local seascape features may contribute to recruitment heterogeneity (McKeown, Hauser, & Shaw, 2017). The Mozambique Channel has a dominant seasonal anticyclonic cell at the northern entrance, as well as a succession of mesoscale anticyclonic and cyclonic eddies along the Mozambique coast (Schouten, de Ruijter, Van Leeuwen, & Ridderinkhof, 2003; Swart, Lutjeharms, Ridderinkhof, & De Ruijter, 2010). Such features have been suggested to contribute to stochastic recruitment/genetic patchiness within the region in a number of species (Bourjea et al., 2007; Hoareau, Boissin, Paulay, & Bruggemann, 2013; Muths et al., 2015) and may contribute to the differentiation among samples from both species within the Mozambique Channel reported here. Even in the absence of genetically isolated source populations, larval cohesion (Selkoe et al., 2006) may enhance (Waples, 2002) and be effectively indistinguishable from sweepstake effects (Turner, Dowling, Marsh, Kesner, & Kelsen, 2007). Berry, England, Marriott, Burridge, and Newman (2012) reported evidence for cohesion among larval and juvenile L. nebulosus but suggested that this was subsequently dissipated by cumulative dispersal of juveniles with no nonrandom cohesion detected by 8 years of age, by which time *L. nebulosus* are reproductively mature. It is possible that the cohesion reported here is similarly transient, in which case the patterns would reflect harvesting of individuals that may not have attained reproductive maturity, a practice that can severely compromise sustainability.

Disentangling the exact roles of recruitment heterogeneity and restricted gene flow will require more sensitive genetic assays and would benefit from analysis of age‐segregated samples (Burford, Carr, & Bernardi, 2011). However, the overall results indicate processes within the region that prevent genetic mixing, at least, on timescales of interest to fishery managers. These contrast with the broad‐scale genetic homogeneity and subtle patchiness reported for *L. nebulosus* in Australian waters (Berry et al., 2012) and implicate regional seascape drivers within the SWIO. In these cases, a spatial “bet‐hedging” approach is advised for marine resource management, including geographical dispersion of marine reserves if they are to be used (Larson & Julian, 1999). The ability to predict population structure across taxa applies directly to the implementation of MPAs. *L. harak* and *L. mahsena* reported similar historical modeled habitat occupancy and phylogeographic patterns, while resolved genetic patchiness points to similar recurrent population processes in both species. Therefore, while we agree that making sweeping predictions from alleged model organisms can be dangerous (Bird et al., 2007), this similarity suggests that the application of information from such models to subsets of taxa may be highly useful in light of finite resources for conservation. Paradoxically, the identification of a cryptic sympatric species assemblage with little (if any) morphological differentiation, that is potentially endemic to the Seychelles, highlights the utility and necessity of genetic approaches to characterize baseline biodiversity in the region before such model‐based methods are employed.

## CONFLICT OF INTEREST

None declared.

## AUTHOR CONTRIBUTIONS

P.W.S. and N.J.M. gained funding for and conceived the project with input from A.J.E.H. and A.T.; data were collected and analyzed by A.J.E.H. and A.T.; and J.P. conducted the ecological niche modeling. A.J.E.H. and N.J.M led the writing of the manuscript, and all authors contributed to editing the manuscript.

## DATA ARCHIVING

All data will be uploaded on DRYAD.
