# Supplementary material for: Cryptic species and parallel genetic structuring in Lethrinid fish: Implications for conservation and management in the southwest Indian Ocean
Source: Ecol Evol. 2018 Jan 24;8(4):2182–95. doi: 10.1002/ece3.3775 (PMC5817149; doi:10.1002/ece3.3775)
Supplement: Supplementary file 1 [file ECE3-8-2182-s001.docx]

**Appendix**

**Appendix S1: PCR-RFLP protocol**


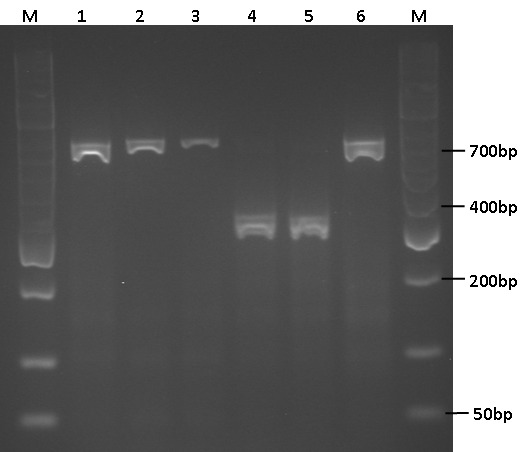


Figure S1: mtDNA gene digest of *Lethrinus mahsena* clades A and B and their associated fragment patterns when separated by gel electrophoresis. *M* molecular weight marker Bioline Hyperladder 50bp; *1* unrestricted PCR product *L. mahsena* clade A; *2-3* gene digest of *L. mahsena* clade A; *4-5* gene digest of *L. mahsena* clade B; *6* unrestricted PCR product *L. mahsena* clade B.

Method: In order to differentiate between the two highly divergent mtDNA COI clades observed in *L. mahsena* (see 3.1), a PCR-RFLP assay was developed. This method allowed for the high throughput screening of all *L. mahsena* individuals, thus permitting direct comparisons of mtDNA and microsatellite genotypes. Sequence alignments of the CO1 gene of both clades of *L. mahsena* were analysed using NEB cutter v2.0 (Vincze et al. 2003) and diagnostic restriction enzyme cleavage sites for the enzyme *FauI* were identified. Species specific COI primers (see methods for PCR protocol) were then used to PCR amplify a 480bp fragment of the CO1 gene. The amplified CO1 fragment was then cut in a subsequent reaction using the enzyme *FauI*. Each reaction contained 5µL of the amplified CO1 sequence, 0.1 µL of *FauI*, and 1µL of enzyme specific buffer and 3.9µL of ddH_2_O. This was then incubated in a thermocycler for 2 hours at 55°C followed by 20 minutes at 65°C. Finally, individual mtDNA clades were assigned by gel electrophoresis (Fig. S1) and microsatellite genotypes assigned to mtDNA clades for subsequent analysis

**Supplementary material**

Table S1: Bioclimatic variables used in species distribution modelling.

**Table S2: Haplotype frequencies for 486bp of *Lethrinus mahsena* COI mtDNA (Haplotypes 4-8 belong to Clade B).**

| **Haplotype** | **MaM** | **SP** | **SM** | **TZ** | **MzP** | **MdB** | **MzM** | **Total** |
| --- | --- | --- | --- | --- | --- | --- | --- | --- |
| **1** | 12 | 0 | 1 | 12 | 1 | 30 | 4 | 33 |
| **2** | 0 | 0 | 1 | 0 | 0 | 0 | 0 | 1 |
| **3** | 0 | 0 | 1 | 0 | 0 | 0 | 0 | 1 |
| **4** | 0 | 0 | 1 | 0 | 0 | 0 | 0 | 1 |
| **5** | 0 | 1 | 2 | 0 | 0 | 0 | 0 | 3 |
| **6** | 0 | 1 | 1 | 0 | 0 | 0 | 0 | 2 |
| **7** | 0 | 6 | 6 | 0 | 0 | 0 | 0 | 12 |
| **8** | 0 | 1 | 0 | 0 | 0 | 0 | 0 | 1 |
| **9** | 0 | 0 | 0 | 1 | 0 | 0 | 0 | 1 |
| **10** | 0 | 0 | 0 | 1 | 1 | 0 | 0 | 2 |
| **11** | 3 | 0 | 0 | 2 | 1 | 0 | 1 | 7 |
| **12** | 2 | 0 | 0 | 2 | 0 | 0 | 1 | 5 |
| **13** | 1 | 0 | 0 | 0 | 0 | 0 | 0 | 1 |
| **14** | 0 | 0 | 0 | 0 | 0 | 2 | 0 | 2 |
| **Total** | 18 | 9 | 13 | 18 | 3 | 5 | 6 | 72 |

Table S3: Haplotype frequencies for 510 bp of *Lethrinus harak* COI mtDNA.

| **Haplotype** | **MaM** | **TZ** | **MzP** | **MdB** | **Total** |
| --- | --- | --- | --- | --- | --- |
| **1** | 6 | 9 | 17 | 28 | 60 |
| **2** | 16 | 7 | 4 | 13 | 40 |
| **3** | 0 | 1 | 0 | 0 | 1 |
| **4** | 0 | 1 | 0 | 0 | 1 |
| **5** | 0 | 0 | 1 | 1 | 2 |
| **Total** | 22 | 18 | 22 | 22 | 104 |

Table S4: Kamura-2-Parameter genetic distances between (below diagonal) and within (on diagonal) putative Lethrinid species, *L. mahsena* (Moz) and *L.* mahsena (India) correspond to GENBANK samples: KJ920117.1, EF609387.1, JF493750.1, JF493751.1 and JF493752.1. Standard errors given in brackets.

|  | ***L.mahsena* CA** | ***L.mahsena* (Moz)** | ***L. mahsena* CB** | ***L.mahsena* (India)** | ***L. atkinsoni*** | ***L. harak*** |
| --- | --- | --- | --- | --- | --- | --- |
| ***L.mahsena* CA** | 0.003 (0.002) |  |  |  |  |  |
| ***L.mahsena* (Moz)** | 0.002 (0.001) | 0.003 (0.002) |  |  |  |  |
| ***L. mahsena* CB** | 0.015 (0.003) | 0.048 (0.010) | 0.002 (0.001) |  |  |  |
| ***L. mahsena* (Ind)** | 0.047 (0.010) | 0.047 (0.010) | 0.004 (0.002) | 0.006 (0.003) |  |  |
| ***L. atkinsoni*** | 0.046 (0.009) | 0.048 (0.010) | 0.040 (0.008) | 0.024 (0.006) | 0.001 (0.001) |  |
| ***L. harak*** | 0.091 (0.014) | 0.091 (0.015) | 0.092 (0.013) | 0.094 (0.015) | 0.100 (0.015) | 0.006 (0.003) |

Table S5: Likelihood of detecting significance (from zero) of a range of *F*_ST_ values based on POWSIM analyses of 486bp mtDNA (COI) for *L. harak* and *L. mahsena* and 10 and 9 microsatellite loci in *Lethrinus mahsena* *Lethrinus harak* respectively. Comparisons were made between average (a) and minimum (m) population sizes for the respective datasets.

|  | **COI** | | | | | | **msat** | | | | | |
| --- | --- | --- | --- | --- | --- | --- | --- | --- | --- | --- | --- | --- |
|  | ***L. harak*** | | | ***L. mahsena*** | | | ***L.harak*** | | | ***L. mahsena*** | | |
|  | **a v a** | **a v m** | **m v m** | **a v a** | **a v m** | **m v m** | **a v a** | **a v m** | **m v m** | **a v a** | **a v m** | **m v m** |
| **F_ST_=0** | 0.038 | 0.024 | 0.035 | 0.04 | 0.026 | 0.013 | 0.049 | 0.035 | 0.043 | 0.047 | 0.028 | 0.008 |
| **F_ST_=0.05** | 0.222 | 0.163 | 0.106 | 0.074 | 0.125 | 0.009 | 1 | 1 | 1 | 0.94 | 0.659 | 0.233 |

Table S6: Microsatellite genetic diversity across 10 loci and 6 populations in *L. mahsena*: microsatellite sample size = N, number of alleles = N_A_, Allelic richness= A_R_, expected heterozygosity = H_E_, observed heterozygosity = H_O_ and probability of deviation from Hardy Weinberg expectations = HWE.

|  |  | **80** | **951** | **90** | **2.33** | **58** | **100** | **96** | **952** | **68** | **75** | **Total** |
| --- | --- | --- | --- | --- | --- | --- | --- | --- | --- | --- | --- | --- |
| **MaM** | **N** | 19 | 19 | 16 | 19 | 17 | 19 | 18 | 19 | 19 | 19 | 18.4 |
|  | **N_A_** | 4 | 3 | 4 | 6 | 7 | 2 | 3 | 6 | 5 | 4 | 4.4 |
|  | **A_R_** | 3.200 | 2.596 | 3.113 | 3.573 | 3.889 | 1.995 | 2.392 | 3.386 | 3.023 | 2.137 | - |
|  | **H_O_** | 0.684 | 0.684 | 0.500 | 0.526 | 0.824 | 0.684 | 0.389 | 0.684 | 0.579 | 0.368 | 0.592 |
|  | **H_E_** | 0.652 | 0.506 | 0.662 | 0.693 | 0.689 | 0.494 | 0.406 | 0.591 | 0.568 | 0.316 | 0.558 |
|  | **F_IS_** | -0.022 | -0.330 | 0.275 | 0.265 | -0.167 | -0.361 | 0.070 | -0.130 | 0.008 | -0.140 | -0.034 |
|  | **HWE** | **0.000** | 0.269 | 0.183 | 0.076 | 0.945 | 0.176 | 0.471 | 0.568 | 0.766 | 1.000 | **0.023** |
| **SM** | **N** | 10 | 9 | 9 | 10 | 9 | 10 | 10 | 10 | 9 | 10 | 9.6 |
|  | **N_A_** | 3 | 2 | 4 | 4 | 2 | 2 | 4 | 4 | 7 | 4 | 3.6 |
|  | **A_R_** | 2.935 | 1.999 | 2.333 | 2.200 | 1.996 | 1.999 | 3.029 | 3.294 | 4.812 | 2.200 | - |
|  | **H_O_** | 0.800 | 0.889 | 0.333 | 0.200 | 0.556 | 0.900 | 0.500 | 0.500 | 0.778 | 0.300 | 0.576 |
|  | **H_E_** | 0.655 | 0.494 | 0.296 | 0.270 | 0.475 | 0.495 | 0.595 | 0.655 | 0.790 | 0.270 | 0.500 |
|  | **F_IS_** | -0.171 | -0.778 | -0.067 | 0.308 | -0.111 | -0.800 | 0.211 | 0.286 | 0.074 | -0.059 | -0.098 |
|  | **HWE** | **0.002** | 0.056 | 1.000 | 0.158 | 1.000 | **0.046** | 0.489 | 0.633 | 0.600 | 1.000 | **0.044** |
| **TZ** | **N** | 19 | 21 | 21 | 20 | 21 | 21 | 21 | 21 | 21 | 21 | 20.7 |
|  | **N_A_** | 2 | 3 | 5 | 5 | 7 | 3 | 3 | 6 | 5 | 5 | 4.4 |
|  | **A_R_** | 1.955 | 2.154 | 3.904 | 3.899 | 4.804 | 2.299 | 1.934 | 2.715 | 2.706 | 2.797 | - |
|  | **H_O_** | 0.474 | 0.667 | 0.714 | 0.500 | 0.714 | 0.476 | 0.190 | 0.429 | 0.571 | 0.619 | 0.535 |
|  | **H_E_** | 0.411 | 0.459 | 0.757 | 0.738 | 0.817 | 0.472 | 0.254 | 0.407 | 0.481 | 0.488 | 0.528 |
|  | **F_IS_** | -0.125 | -0.432 | 0.081 | 0.345 | 0.150 | 0.015 | 0.273 | -0.029 | -0.165 | -0.247 | 0.011 |
|  | **HWE** | 1.000 | 0.077 | 0.853 | **0.018** | 0.271 | 1.000 | 0.173 | 0.769 | 0.808 | 0.891 | 0.412 |
| **MzP** | **N** | 7 | 7 | 7 | 6 | 7 | 7 | 7 | 7 | 7 | 7 | 6.9 |
|  | **N_A_** | 2 | 3 | 2 | 2 | 4 | 3 | 4 | 5 | 4 | 2 | 3.1 |
|  | **A_R_** | 1.985 | 2.407 | 1.835 | 1.909 | 2.978 | 2.820 | 3.501 | 4.294 | 3.392 | 1.571 | - |
|  | **H_O_** | 0.571 | 0.429 | 0.000 | 0.000 | 0.429 | 0.857 | 0.857 | 0.857 | 0.429 | 0.143 | 0.457 |
|  | **H_E_** | 0.408 | 0.357 | 0.245 | 0.278 | 0.459 | 0.571 | 0.684 | 0.755 | 0.643 | 0.133 | 0.453 |
|  | **F_IS_** | -0.333 | -0.125 | 1.000 | 1.000 | 0.143 | -0.440 | -0.180 | -0.059 | 0.400 | - | 0.070 |
|  | **HWE** | 1.000 | 1.000 | 0.077 | 0.091 | 0.441 | 0.478 | 0.712 | 0.471 | **0.040** | - | 0.246 |
| **MzM** | **N** | 8 | 10 | 10 | 8 | 10 | 10 | 10 | 10 | 10 | 9 | 9.5 |
|  | **N_A_** | 3 | 3 | 3 | 4 | 8 | 3 | 3 | 5 | 3 | 1 | 3.6 |
|  | **A_R_** | 2.754 | 2.349 | 2.305 | 2.987 | 5.412 | 2.602 | 2.602 | 3.954 | 2.305 | 1.000 | - |
|  | **H_O_** | 0.250 | 0.400 | 0.400 | 0.250 | 1.000 | 0.600 | 0.700 | 0.700 | 0.200 | 0.000 | 0.450 |
|  | **H_E_** | 0.570 | 0.445 | 0.340 | 0.578 | 0.840 | 0.505 | 0.505 | 0.745 | 0.340 | 0.000 | 0.487 |
|  | **F_IS_** | 0.606 | 0.153 | -0.125 | 0.611 | -0.133 | -0.137 | -3.404 | 0.113 | 0.455 | - | 0.133 |
|  | **HWE** | **0.021** | 1.000 | 1.000 | **0.021** | 0.733 | 1.000 | 0.505 | **0.035** | 0.108 | - | 0.053 |
| **MDB** | **N** | 5 | 5 | 5 | 4 | 5 | 5 | 5 | 5 | 5 | 5 | 4.9 |
|  | **N_A_** | 2 | 2 | 2 | 2 | 5 | 2 | 1 | 2 | 3 | 2 | 2.3 |
|  | **A_R_** | 2.000 | 2.000 | 2.000 | 2.000 | 4.578 | 2.000 | 1.000 | 1.978 | 2.800 | 1.800 | - |
|  | **H_O_** | 0.600 | 0.800 | 0.600 | 0.500 | 1.000 | 0.800 | 0.000 | 0.400 | 0.400 | 0.200 | 0.530 |
|  | **H_E_** | 0.420 | 0.480 | 0.420 | 0.375 | 0.760 | 0.480 | 0.000 | 0.320 | 0.580 | 0.180 | 0.402 |
|  | **F_IS_** | -0.333 | -0.600 | -0.333 | -0.200 | -0.212 | -0.600 | - | -0.143 | 0.407 | - | -0.214 |
|  | **HWE** | 1.000 | 0.429 | 1.000 | 1.000 | 0.848 | 0.429 | - | 1.000 | 0.365 | - | 0.991 |
| **Total** | **N** | 11.333 | 11.833 | 11.333 | 11.167 | 11.500 | 12.000 | 11.833 | 12.000 | 11.833 | 11.833 | 11.667 |
|  | **N_A_** | 2.667 | 2.667 | 3.333 | 3.833 | 5.500 | 2.500 | 3.000 | 4.667 | 4.500 | 3.000 | 3.567 |
|  | **A_R_** | 3.146 | 2.896 | 3.173 | 4.045 | 4.589 | 2.286 | 2.860 | 3.674 | 3.634 | 2.189 | - |
|  | **H_O_** | 0.563 | 0.645 | 0.425 | 0.329 | 0.754 | 0.720 | 0.439 | 0.595 | 0.493 | 0.272 | 0.523 |
|  | **H_E_** | 0.520 | 0.457 | 0.453 | 0.488 | 0.673 | 0.503 | 0.407 | 0.579 | 0.567 | 0.231 | 0.488 |
|  | **F_IS_** | -0.084 | -0.411 | 0.064 | 0.326 | -0.119 | -0.431 | -0.079 | -0.028 | 0.131 | -0.176 | -0.081 |
|  | **HWE** | **0.000** | 0.229 | 0.716 | **0.004** | 0.947 | 0.382 | 0.581 | 0.546 | 0.247 | 1.000 | **0.025** |

Table S7: Microsatellite genetic diversity across 9 loci and 4 populations in *L. harak*: microsatellite sample size = N, number of alleles = N_A_, Allelic richness= A_R_, expected heterozygosity = H_E_, observed heterozygosity = H_O_ and probability of deviation from Hardy Weinberg expectations = HWE.

|  |  | **80** | **90** | **2.33** | **58** | **100** | **96** | **952** | **68** | **75** | **Total** |
| --- | --- | --- | --- | --- | --- | --- | --- | --- | --- | --- | --- |
| **MaM** | **N** | 39 | 41 | 42 | 37 | 43 | 43 | 43 | 43 | 43 | 41.556 |
|  | **N_A_** | 2 | 2 | 6 | 5 | 3 | 3 | 7 | 6 | 2 | 4.000 |
|  | **A_R_** | 2.000 | 2.000 | 5.524 | 5.000 | 2.858 | 2.982 | 6.858 | 5.840 | 2.000 |  |
|  | **H_O_** | 0.487 | 0.341 | 0.310 | 0.595 | 0.093 | 0.837 | 0.860 | 0.605 | 0.512 | 0.516 |
|  | **H_E_** | 0.369 | 0.283 | 0.383 | 0.716 | 0.090 | 0.505 | 0.781 | 0.494 | 0.381 | 0.445 |
|  | **F_IS_** | -0.310 | -0.194 | 0.203 | 0.183 | -0.028 | -0.651 | -0.091 | -0.212 | -0.333 | -0.148 |
|  | **HWE** | 0.080 | 0.573 | **0.043** | **0.001** | 1.000 | **0.000** | 0.097 | 0.631 | **0.040** | **0.000** |
| **TZ** | **N** | 60 | 60 | 60 | 57 | 57 | 55 | 58 | 57 | 59 | 58.111 |
|  | **N_A_** | 2 | 2 | 4 | 5 | 4 | 3 | 7 | 8 | 2 | 4.111 |
|  | **A_R_** | 2.000 | 1.997 | 3.327 | 4.999 | 4.000 | 3.000 | 6.998 | 7.703 | 2.000 |  |
|  | **H_O_** | 0.333 | 0.100 | 0.050 | 0.544 | 0.596 | 0.545 | 0.690 | 0.667 | 0.288 | 0.424 |
|  | **H_E_** | 0.278 | 0.095 | 0.081 | 0.681 | 0.525 | 0.444 | 0.829 | 0.620 | 0.247 | 0.422 |
|  | **F_IS_** | -0.192 | -0.044 | 0.390 | 0.210 | -0.128 | -0.219 | 0.177 | -0.066 | -0.160 | 0.005 |
|  | **HWE** | 0.340 | 1.000 | **0.001** | **0.001** | 0.698 | 0.167 | **0.013** | 0.465 | 0.588 | **0.000** |
| **MzP** | **N** | 44 | 44 | 43 | 43 | 44 | 42 | 43 | 43 | 44 | 43.333 |
|  | **N_A_** | 3 | 2 | 2 | 4 | 4 | 3 | 7 | 8 | 2 | 3.889 |
|  | **A_R_** | 3.000 | 1.997 | 1.860 | 3.982 | 3.682 | 3.000 | 6.860 | 7.842 | 2.000 |  |
|  | **H_O_** | 0.205 | 0.068 | 0.023 | 0.349 | 0.455 | 0.738 | 0.744 | 0.651 | 0.455 | 0.410 |
|  | **H_E_** | 0.362 | 0.066 | 0.023 | 0.530 | 0.361 | 0.602 | 0.771 | 0.682 | 0.351 | 0.416 |
|  | **F_IS_** | 0.444 | -0.024 | - | 0.353 | -0.249 | -0.215 | 0.047 | 0.057 | -0.284 | 0.028 |
|  | **HWE** | **0.000** | 1.000 | - | **0.003** | 0.485 | **0.036** | 0.423 | 0.109 | 0.086 | **0.000** |
| **MDB** | **N** | 97 | 99 | 99 | 97 | 100 | 94 | 96 | 94 | 96 | 96.889 |
|  | **N_A_** | 3 | 3 | 4 | 6 | 5 | 4 | 8 | 8 | 2 | 4.778 |
|  | **A_R_** | 2.381 | 2.929 | 3.201 | 5.147 | 3.726 | 3.394 | 7.811 | 7.318 | 2.000 |  |
|  | **H_O_** | 0.340 | 0.141 | 0.091 | 0.588 | 0.280 | 0.564 | 0.750 | 0.755 | 0.229 | 0.415 |
|  | **H_E_** | 0.284 | 0.134 | 0.125 | 0.643 | 0.251 | 0.560 | 0.822 | 0.717 | 0.249 | 0.421 |
|  | **F_IS_** | -0.193 | -0.051 | 0.277 | 0.091 | -0.110 | -0.001 | 0.093 | -0.049 | 0.085 | 0.017 |
|  | **HWE** | 0.130 | 1.000 | **0.000** | **0.000** | 0.887 | 0.991 | 0.104 | 0.067 | 0.412 | **0.000** |
| **Total** | **N** | 60.000 | 61.000 | 61.000 | 58.500 | 61.000 | 58.500 | 60.000 | 59.250 | 60.500 | 59.972 |
|  | **N_A_** | 2.500 | 2.250 | 4.000 | 5.000 | 4.000 | 3.250 | 7.250 | 7.500 | 2.000 | 4.194 |
|  | **A_R_** | 2.970 | 2.680 | 4.141 | 5.096 | 4.125 | 3.158 | 7.738 | 7.764 | 2.000 |  |
|  | **H_O_** | 0.341 | 0.163 | 0.118 | 0.519 | 0.356 | 0.671 | 0.761 | 0.669 | 0.371 | 0.441 |
|  | **H_E_** | 0.323 | 0.144 | 0.153 | 0.642 | 0.307 | 0.528 | 0.801 | 0.628 | 0.307 | 0.426 |
|  | **F_IS_** | -0.057 | -0.127 | 0.225 | 0.193 | -0.161 | -0.271 | 0.050 | -0.066 | -0.208 | -0.047 |
|  | **HWE** | **0.000** | 0.997 | **0.000** | **0.000** | 0.966 | **0.000** | **0.012** | 0.138 | 0.078 | **0.000** |

Figure S1: Assessment of number of populations of A) *Lethrinus mahsena* and B) *Lethrinus harak* across the SWIO, estimated through Bayesian admixture implemented in BAPS.


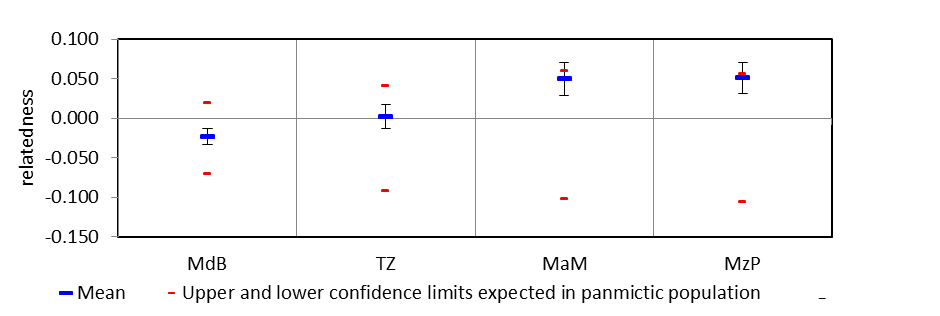


Figure S2: Relatedness (r_qg_) for *Lethrinus harak* samples across the SWIO.


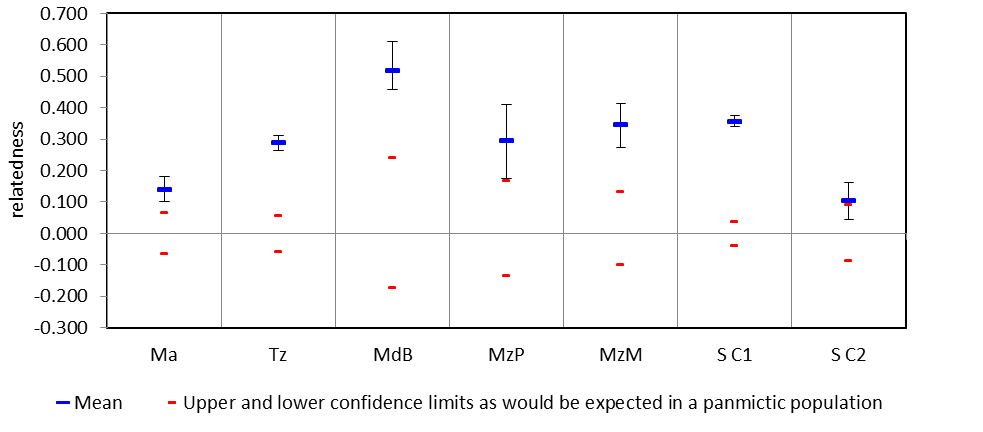
 Figure S3: Relatedness (r_qg_) for *Lethrinus mahsena* samples across the SWIO, Seychelles islands are pooled and separated by mtDNA clade A (S C1) and clade B (S C2).


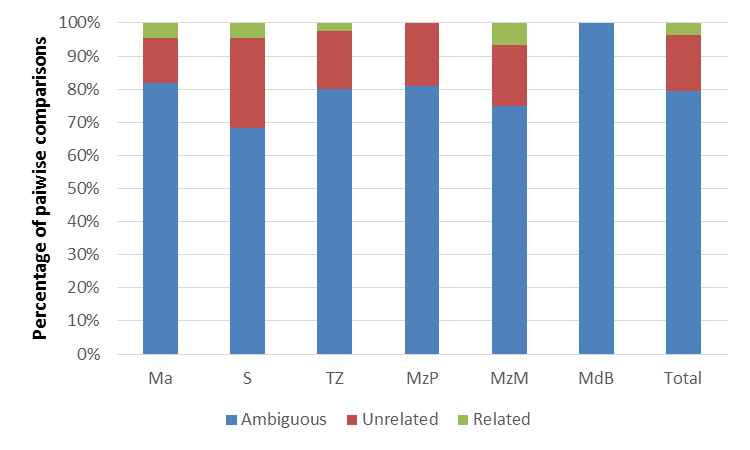


Figure S4: Percentage of relationships between pairs of individuals of *L. mahsena* that fell within 3 relatedness classifications: (a). unrelated (classification as U only), (b) related (classification as any combination of Half Sibling, Full Sibling, Parent-Offspring but not Unrelated), (c) ambiguous (classification as Unrelated as well as some related state). Based on 10 microsatellite loci.


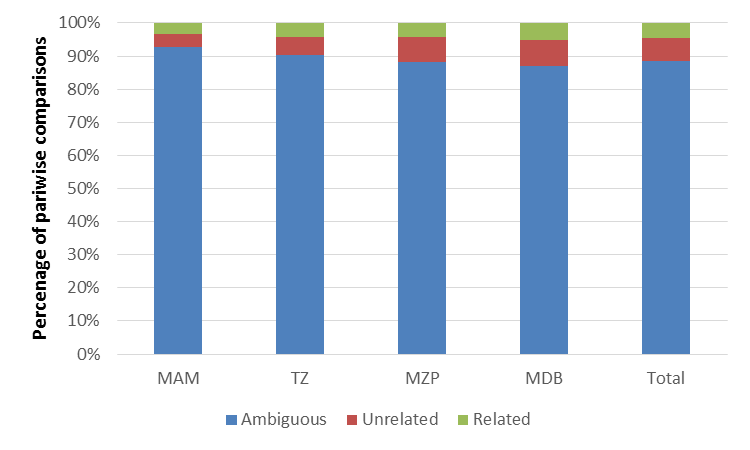


Figure S5: Percentage of relationships between pairs of individuals of *L. harak* that fell within 3 relatedness classifications: (a). unrelated (classification as U only), (b) related (classification as any combination of Half Sibling, Full Sibling, Parent-Offspring but not Unrelated), (c) ambiguous (classification as Unrelated as well as some related state). Based on 9 microsatellite loci.
